# Exploring Antioxidant Synergism Mechanism of 3D Printing Based Shanyao–Fuling–Yiyiren Decoction via Fuzzy Mathematical Method, Network Pharmacology, and In Vitro Experimental Validation

**DOI:** 10.1002/fsn3.70349

**Published:** 2025-05-30

**Authors:** Haoran Fan, Cai You, Chenxi Ren, Xiaoxiao Liu, Yining Feng, Ganghui Chu, Abdulla Yusuf, Chunbo Liu, Liemin Ruan, Jia Xu, Tianzhu Guan

**Affiliations:** ^1^ College of Tourism and Culinary Science Yangzhou University Yangzhou Jiangsu China; ^2^ School of Medicine Ningbo University Ningbo China; ^3^ School of Food Science and Engineering Yangzhou University Yangzhou China; ^4^ College of Chemistry and Environmental Science, Laboratory of Xinjiang Native Medicinal and Edible Plant Resources Chemistry Kashi University Kashi China; ^5^ The First Affiliated Hospital of Ningbo University Ningbo University Ningbo China

**Keywords:** 3D printing food, AKT1/GSK3β/HIF1α pathway, fuzzy mathematical model, network pharmacology, Shanyao–Fuling–Yiyiren (SFY)‐based polyherbal formulation

## Abstract

This study demonstrates the antioxidative stress potential of Shanyao–Fuling–Yiyiren (SFY) decoction—a Chinese polyherbal formulation derived from Si Fang decoction—by establishing a systematic framework that integrates network pharmacology, molecular docking, in vitro synergy assays, cellular experiments, and 3D printing. Despite its long traditional use, the molecular and cellular mechanisms underlying its antioxidative effects remain unclear, and its formulations are based more on empirical methods than on systematic design. To fill this gap, a fuzzy mathematical model was used to select the optimal polyherbal combination. A central composite circumscribed design determined that a Shanyao:Fuling:Yiyiren ratio of 2:2:1 maximized radical scavenging, with a strong correlation (*R* ^2^ = 0.9665) between antioxidant activity and the combination index. Furthermore, network pharmacology, molecular docking, and cell‐based assays jointly confirm the AKT1/GSK3β/HIF1α pathway plays a crucial role in preventing the antioxidant effects of SFY. Finally, the development of 3D printing SFY‐inks with the optimized shape fidelity suggests promising applications for both nutraceuticals and hepatocellular carcinoma diagnosis. Overall, the results prove that 3D printing SFY‐based polyherbal formulation with promising antioxidant potential and maximum synergism may indeed be a potential source of preventing oxidant damages in pharmaceutical and food industries.

## Graphical abstract

SFY‐based polyherbal formulation was selected based on fuzzy mathematical model. The best combinations with synergistic effect were optimized and identified by central composite circumscribed design. Network pharmacology and molecular docking‐based approach revealed that the potential mechanisms of decoction against oxidant damage via multicomponent, multitarget, and multipathway. Decoction presented ideal antioxidative protection activity against H_2_O_2_ damage. Decoction‐based inks have great practical printability and 3D printing performance for potential application in functional food production.

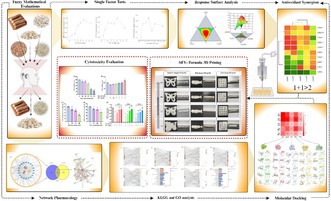

## 1 Introduction

About 95% of humans worldwide stay up late at night, but handling jet lag can disrupt circadian rhythms, which is considered a stress state related to many diseases, including metabolic disorders and liver cancer. As a vital organ in charge of detoxification, the liver is very sensitive to damages from physiological stress, resulting in life‐threatening conditions (e.g., liver injury) with a high mortality rate and poor prognosis. Among the many causes of liver injury, uninterrupted production of reactive oxygen species (ROS) from both endogenous and exogenous origins has received much attention, including hydroxyl radical (OH˙), hydrogen peroxide (H_2_O_2_), hydroxyl ion (OH^−^), singlet oxygen (^1^O_2_), superoxide anion (O_2_˙^−^), and ozone (O_3_) (Chen et al. 2020). Studies have demonstrated a connection between prolonged exposure to ROS and liver diseases (Prieto and Monsalve 2017). The elevated levels of ROS are intracellular signaling factors that remarkably enhance hepatic stellate cell activation and extracellular matrix generation amid liver injuries. Additionally, ROS activates significant oncogenic pathways leading to hepatocarcinogenesis, such as extracellular signal‐regulated kinase, protein kinase B, c‐Jun N‐terminal kinase, hypoxia‐inducible factor (HIF), microtubule‐related protein kinase, and intensified cellular DNA mutations (Hammouda et al. 2020). Concurrently, concerns about the adverse effects of synthetic antioxidants in foods have diverted more attention to novel sources of natural antioxidants from the safety aspect. Given the critical role of oxidative stress in liver disease occurrence, the role of phospholipid peroxidation in liver injuries shall be urgently explored. Against this backdrop, we hypothesize that phospholipid peroxidation represents a key mechanistic link between circadian disruption and stress‐related liver injuries.

Emerging evidence has described that traditional medicinal herbs potentially contribute to preventing oxidative stress‐related chronic diseases (Allison et al. 2025; Ashraf et al. 2024). These herbs have been applied conventionally for millennia by many cultures as medicine, flavoring reagents, and even food preservatives, and are basically cheap and available for poor populations. Researchers have tested their antioxidant abilities and potential replacements of synthetic additives in protecting food and cosmetic products from oxidative damages. Specifically, Shanyao (*Rhizoma Dioscoreae*), Fuling (*Poria cocos (Schw.) Wolf*.), and Yiyiren (*Coicis Semen*) are widely used in traditional medicine, owing to their potent antioxidant properties. Shanyao in the crude form has long been used as a spice, dietary supplement, and a constituent of many traditional Asian medicines (Luo et al. 2024). Shanyao contains curcumin, which has these pharmacological abilities owing to its basic beneficial antioxidant, anti‐inflammatory, antibacterial, and anticancer abilities (Alam et al. 2024). Fuling has diverse pharmacological activities against rheumatoid arthritis, type II diabetes, multiple sclerosis, atherosclerosis, Alzheimer's disease, and other chronic diseases (Guo et al. 2025). Free radicals, which are key stimuli for carcinogenesis, can be inhibited by Fuling from modulating lipid peroxidation of membranes or oxidative DNA harms. As for the antioxidant role, Yiyiren is proved to effectively scavenge diverse risky free radicals, including ROS, NO_2_ radicals, O_2_˙^−^, and OH˙ (Zhang et al. 2024).

Regardless of the abundant research, observations about the antioxidant synergism in mixtures are still deficient. Moreover, recent studies have raised significant controversy regarding the standardization of such evaluations, reflecting a wider gap in understanding the underlying mechanisms of antioxidant synergism (Cnudde et al. 2024; Eawsakul and Bunluepuech 2024; Shen et al. 2025). This lack of consensus has led to speculation and explanations in the literature, many of which lack empirical verification. In view of antioxidant synergism effects, the mixing of antioxidant extracts may induce a synergism to generate a better antioxidant effect than the sum produced by single extracts (additives) (Eawsakul and Bunluepuech 2024; Kurnia et al. 2025). Moreover, the ratio and type of individual herb extracts are pivotal in deciding the chemo‐preventive potential or antioxidant capacity in a health‐benefiting herb combination. Thus, the regulation of antioxidant properties shall be investigated according to the proportions of herbs in a mixture that can be used to develop functional foods and pharmaceutical products at different contents. For instance, the combination of *Osmanthus fragrans* flower extract with four types of tea (Longjing, Tieguanyin, black, or Pu'er Tea) can synergistically scavenge 2,2‐diphenyl‐1‐picrylhydrazyl free radicals (Mao et al. 2017), demonstrating how specific combinations can enhance antioxidant performance beyond individual components.

To our knowledge, though the antioxidant activities of Shanyao, Fuling, and Yiyiren have been extensively reported, there is little research on the bio‐effects of extracts combined with bioactive compounds, which are believed to improve the antioxidant benefit of free radical scavenging. Therefore, this study was aimed primarily to explore the antioxidant interaction among Shanyao, Fuling, and Yiyiren at various proportions using response surface methodology (RSM) and the combination index. On the basis of synergism and optimization of the decoction process, we simultaneously conducted network pharmacology and molecular docking experiments to elucidate the mechanisms of the Shanyao, Fuling, and Yiyiren compound (SFY) binding with AKT1, GSK3B, TP53, HIF1A, and PTGS2‐related targets. Then whether SFY can mitigate oxidative stress via the AKT1/GSK3β/HIF1α antioxidant system was evaluated, aiming to provide insight into the intervention of H_2_O_2_‐induced oxidative injuries. Finally, a decoction‐based broad spectrum of edible inks was selected and developed for a 3D printing ink in a balanced diet. Collectively, the present study provides a molecular basis for using SFY as a promising antioxidant in the future.

## 2 Materials and Methods

### 2.1 Reagents and Materials

Shanyao, Fuling, and Yiyiren were obtained from a local market of Yangzhou. 2,2′‐Azino‐bis (3‐ethylbenzothiazoline‐6‐sulphonic acid) (ABTS) and 2,4,6‐tris (2‐pyridyl)‐1,3,5‐triazine (TPTZ) were made in Aldrich‐Sigma (St. Louis, MO, USA). H_2_O_2_ (30%), chloroform, anhydrous ethanol, and isopropanol were bought from Sinopharm Chemical Reagent Co. Ltd. (China). TRIzol was obtained from Thermo Fisher Scientific (USA). A real‐time fluorescence quantitative PCR system (qRT‐PCR) and a reverse transcriptional kit were purchased from TransGen Biotech (China). Superoxide dismutase (SOD) and catalase (CAT) activity detection kits were obtained from Beyotime Biotechnology and Grace Biotechnology (both China), respectively. Dimethyl sulfoxide (DMSO), ethylene diamine tetraacetic acid (EDTA), phosphate buffer solution (PBS) phosphate dry powder, and 3‐(4,5‐dimethylthiazol‐2‐yl)‐2,5‐diphenyltetrazolium bromide (MTT) were offered by Beijing Solarbio (China). All other agents were analytically pure.

### 2.2 Optimization and Preparation of Formulations of Decoction

The preparations of medicinal and edible herbals were determined according to the method from Guan et al. (2021). Based on a modified classic folk recipe, Sifang Tang, Shanyao, Fuling, and Yiyiren were selected, cleaned, and air‐dried at 60°C overnight. Then the dried materials were filtrated through an 80‐mesh sieve and kept in an airtight container at ambient temperature until used. With the solid/liquid ratio at 1:20, the three types of herbal powders were randomly combined and thoroughly mixed in an RK103H ultrasonic bath (BANDE‐LIN SONOREX, Germany) at 80 kHz. After 4 h of extraction, the extracts were centrifuged at 4500 rpm for 10 min. The final sensory test samples were obtained by mixing the three extracts.

### 2.3 Fuzzy Mathematical‐Based Sensory Evaluation

Sensory evaluation was carried out according to the procedure from Jaya and Das (2003). Briefly, 10 reportedly healthy, nonsmoking potential evaluators from the staff and students of the College of Tourism and Culinary Science, Yangzhou University were selected at a 60% success rate in triangle tests and enrolled to evaluate the color, odor, taste, and texture of sensory test samples. The testing was finished in the laboratory as per regulations in ASTM MNL‐26 (1996). The panelists each sensorily analyzed 10 samples. The tested samples were categorized into four distinct grades: excellent, good, fair, and poor, which reflected their distinctiveness. The criteria for sensory evaluation were listed in Table S1, and the voting outcomes for each criterion were recorded in Table 1.

**TABLE 1:** Sensory evaluation indexes of polyherbal formulations.

Num.	Sample	Color				Odor				Taste				Texture			
		V_1_	V_2_	V_3_	V_4_	V_1_	V_2_	V_3_	V_4_	V_1_	V_2_	V_3_	V_4_	V_1_	V_2_	V_3_	V_4_
*T*_1_	*Euryale ferox* ; Lotus seed; Shanyao	4	2	4	0	3	4	2	1	3	5	1	1	2	6	2	0
*T*_2_	*Euryale ferox* ; Lotus seed; Yiyiren	3	3	4	0	2	4	4	0	6	6	1	0	1	3	5	1
*T*_3_	*Euryale ferox* ; Lotus seed; Fuling	0	4	3	3	0	1	4	5	1	5	3	1	5	3	2	0
*T*_4_	Lotus seed; Shanyao; Yiyiren	5	2	2	1	4	2	2	2	2	4	4	0	3	4	2	1
*T*_5_	Lotus seed; Shanyao; Fuling	4	5	1	0	5	3	1	1	5	5	0	0	4	4	2	0
*T*_6_	Shanyao; Yiyiren; Fuling	6	4	0	0	7	2	1	0	7	3	0	0	3	4	1	2
*T*_7_	*Euryale ferox* ; Shanyao; Yiyiren	4	4	2	0	6	3	1	0	5	4	1	0	2	8	0	0
*T*_8_	*Euryale ferox* ; Shanyao; Fuling	2	1	4	3	3	4	2	1	3	7	0	0	0	6	4	0
*T*_9_	*Euryale ferox* ; Yiyiren; Fuling	3	5	1	1	2	8	0	0	7	1	1	1	5	3	1	1
*T*_10_	Lotus seed; Yiyiren; Fuling	1	6	1	2	5	3	2	0	4	4	2	0	2	5	2	1

Fuzzy mathematical sensory evaluation was integrated into food sensory assessment to quantify evaluation factors, substantially mitigate the influence of personal bias, and produce more accurate and scientific scoring results (Ranneh et al. 2021). In detail, a set of sensory factors for the prepared solutions was defined as U, and a set of grades as V. U comprises color (U_1_), odor (U_2_), taste (U_3_), and texture (U_4_), and V includes excellent (V_1_), good (V_2_), average (V_3_), and poor (V_4_). The relative weight of each sensory factor in the overall flavor profile was determined using the fuzzy binary comparative decision method. Ten sensory evaluators compared these factors pairwise. A factor deemed important was given 1 point, whereas those considered less important were assigned 0 point. The total score of each sensory factor divided by the maximum score of 100 was used as the weight of this sensory factor (Table S2). Based on the evaluators' scoring on the importance of color, odor, taste, and texture in the overall sensory evaluation, the distribution of weights among these factors is *X* = {color, odor, taste, texture} = {0.24, 0.28, 0.33, 0.15}.

### 2.4 Determination of ABTS Radical (ABTS^+^) Scavenging Capacity

The ABTS^+^ scavenging ability was tested following the method from Guan, Li, et al. (2024) with modifications. Briefly, an ABTS^+^ stock solution was prepared by mixing 5 mL of 7 mM ABTS and 5 mL of 2.45 mM potassium persulfate and put for 12–16 h at room temperature (RT) without light. Then the mixture was diluted using ultrapure water until the absorbance at 405 nm was 1.4, forming an ABTS working solution. Gradient diluted extracts or ascorbic acid (0.5 mL) were blended with 0.5 mL of the ABTS working solution and stood for 30 min in the dark at RT. Then the absorbance at 734 nm was recorded. The percent of ABTS^+^ scavenging activity of the extracts was computed using Equation (1):

(1) $$\text{ABTS}\;\left(\%\right)=\left[1-\left(A_{1}-A_{2}\right)/A_{0}\right]\times 100\%$$

where *A*_1_, *A*_2_, and *A*_0_ are the absorbance of the mixture of ABTS^+^ and the sample solution, the mixture of ABTS^+^ and the control sample, and the mixture of ABTS^+^ and deionized water, respectively.

### 2.5 Experiment Design and Mixture Optimization

Mixture design, a special type of RSM, was used to optimize the composition of herbal mixtures and test the interactive effect between components. Based on Section 2.3, the polyherbal formulations for further optimization consisted of Shanyao, Fuling, and Yiyiren. In total, 16 formulations were formed and the responses were analyzed using DesignExpert 13 (Minitab Inc., State College, PA, USA). The layout of the herbal formulations is shown in Table 2. The dependent variable was in vitro ABTS antioxidant ability. The canonical model of was used for each response after adjustment based on the testing data. Linear, quadratic, and special cubic models were tested to determine regression coefficients, which were kept only at the significant level. Data were refitted to obtain the final model for each index. The adequacy and goodness of fitting for each model were statistically analyzed and fitted to a second‐order polynomial regression model involving the coefficients of linear, quadratic, and interactive terms. For validation, the optimal formulation was examined in triplicate and expressed as mean ± standard deviation. Analysis of variance (ANOVA) was conducted to calculate the model significance and suitability of the factors and interactions. The formulations with highly desirable functions were chosen for further analysis.

**TABLE 2:** Mixture design experimental arrangement and results.

Standard order	Factor A (Fuling, g)	Factor B (Shanyao, g)	Factor C (Yiyiren, g)	Response
				ABTS^+^ radical scavenging capacity (%)
1	0.75	0.55	0.40	41.30 ± 0.73
2	0.65	0.75	0.30	56.98 ± 0.41
3	0.65	0.65	0.40	54.35 ± 0.98
4	0.75	0.62	0.33	50.76 ± 0.47
5	0.65	0.65	0.40	61.18 ± 0.63
6	0.75	0.65	0.30	55.61 ± 0.24
7	0.65	0.75	0.30	53.13 ± 0.30
8	0.72	0.62	0.37	61.32 ± 1.09
9	0.65	0.75	0.30	54.63 ± 0.38
10	0.75	0.75	0.20	46.27 ± 0.29
11	0.55	0.75	0.40	61.15 ± 0.66
12	0.62	0.72	0.37	67.47 ± 0.68
13	0.55	0.75	0.40	57.46 ± 0.34
14	0.75	0.68	0.27	53.50 ± 0.64
15	0.68	0.68	0.33	67.60 ± 0.60
16	0.65	0.65	0.40	54.94 ± 0.21

### 2.6 Determination of Antioxidant Synergism

Based on the Chou–Talalay combined drug theory, the synergistic antioxidant effects of different combinations were explored (Chou 2018). Specifically, antioxidant synergism was determined as per the sum of IC_50_ from seven concentrations based on RSM to generate a total of 16 solution combinations. The absorbance of the solution combination was then detected to determine the percent of ABTS^+^ scavenging capacity. To quantify the synergistic, additive, or antagonistic impact of the combinations of decoction, the testing data were converted to the combination index (CI):

$$\mathrm{CI}=\left(\mathrm{MCa}/\mathrm{SCa}\right)+\left(\mathrm{MCb}/\mathrm{SCb}\right)$$

where MCa and MCb are the concentrations of compounds A and B in the mixture to achieve 50% antioxidant activity respectively; SCa and SCb are the EC_50_ of the single compounds A and B, respectively. CI < 1, = 1, or > 1 implies a synergistic, additive, or antagonistic effect, respectively.

### 2.7 Identification of Core Ingredients, Potential Targets of Decoction, and Network Construction

The active constituents and related targets in SFY‐based polyherbal formulations were cited from Chinese Medicine System Pharmacology Database (https://old.tcmsp‐e.com/tcmsp.php) with pharmacokinetic parameters (oral bioavailability [OB] ≥ 30% and drug‐like activity [DL] ≥ 0.18). Then the target names of the corresponding core active ingredients were turned into a unified format with *Homo sapiens* via Unified Protein Database (https://www.uniprot.org/). In addition, the potential antioxidant associated targets were gathered from GeneCards (http://www.genecards.org/), Herb 2.0 (http://www.disgenet.org/search), and OMIM (https://omim.org/). Then duplicates were removed after the targets were merged from the databases above. The targets of antioxidants and SFY‐based polyherbal formulations and their common targets were acquired via Venny 2.1.0. To systematically examine the network, the “herb‐compound‐common target” network was imported and constructed via Cytoscape 3.9.1.

### 2.8 Protein–Protein Interaction (PPI) Network Construction, Topology Analysis, and GO/KEGG Enrichment Analysis of Core Targets

To investigate the core targets and their interactions against oxidant damages, the overlapping targets between SFY‐based polyherbal formulations and oxidant damages were examined using the STRING network platform (https://stringdb.org/). Furthermore, a PPI network was created at a confidence level > 0.4 and with the “ *Homo sapiens* ” filter (Ren et al. 2024). The network was further analyzed in Cytoscape 3.9.1 using MCODE to identify the associations between hub genes and network clusters with specific parameters. Core clusters were then identified based on degree centrality. GO and KEGG pathway enrichment analyses of the core targets were done using David 6.8 (https://david.ncifcrf.gov/) to explore specific mechanisms and signaling pathways. Enrichment results with *p* and *q* < 0.05 were visualized using bar and bubble charts on the online tool imageGP.

### 2.9 Molecular Docking

Molecular docking, an increasingly important computational tool for exploring the behaviors of biomacromolecule complexes, was conducted as per the approach of Guan et al. (2023) with modifications. Based on the degrees from Section 2.8, the top targets and core ingredients from the SFY‐based polyherbal formulations were chosen for affinity calculations. The 3D structures of AKT1, GSK3B, TP53, HIF1A, and PTGS2 were cited and constructed via AutoDockTools 1.5.6. Polar hydrogens were added to demand charges after water was removed. The smallest binding energy was computed on AutoDock Vina. Finally, the binding details were illustrated on PyMOL and Discovery Studio 2018.

### 2.10 Cytotoxicity Evaluation and Antioxidant Enzyme Activity Analysis

The BNLCL.2 mouse embryonic liver cell line from Meilun Biotechnology Co. Ltd. (Dalian, China) was cultured in high‐glucose DMEM added with 10% FBS and 1% P/S at 37°C with 5% CO_2_. Trypsin was used for cell passage. For H_2_O_2_ cytotoxicity assessment, the BNLCL.2 cells (1 × 10^4^ cells/well) were planted in 96‐well plates and processed with 0, 200, 400, 600, 800, or 1000 μmol/L H_2_O_2_ for 24 h. To evaluate the protective effect of SFY, the cells were pretreated with 600 μM H_2_O_2_ for 4 h, and co‐cultured with 0.5, 1, 2, 4, or 8 mg/mL SFY for 24 h. In MTT assays, each well was added with 100 μL of 5 mg/mL MTT, incubated for 4 h, and then the formazan crystals were solubilized with DMSO. The absorbance at 490 nm was recorded using a microplate reader (Flefel et al. 2019). Additionally, BNLCL.2 cells were pretreated with H_2_O_2_ or SFY at specific concentrations. Then CAT and SOD activities were assessed using commercial kits (Cat. no. S0101S, G0105W48) as instructed by the manufacturer, and were detected with colorimetry and the xanthine oxidase method, respectively (Ding et al. 2020).

### 2.11 RNA Extraction and qRT‐PCR Analysis

The BNLCL.2 cells were incubated in 6‐well plates for 24 h, pretreated with 600 μM H_2_O_2_ for 4 h, and exposed to SFY seeds (0, 2, 4, and 8 mg/mL) for 24 h. The treated BNLCL.2 cells were collected. Total RNA was extracted from the BNLCL.2 cells using a TRIzol reagent, and reverse transcribed to cDNA using a first‐strand cDNA synthesis SuperMix kit for qPCR (gDNA digester plus) from TransGen Biotech (China). Real‐time PCR was conducted with fast SYBR green master mix (Wang et al. 2023). The quantities of transcripts were standardized to that of GAPDH. The primers (Sangon Biotech, China) were presented in Table S3.

### 2.12 3D Printing Ink Preparation and Printability Assessment

3D printing inks were prepared and assessed in terms of printability by forming different structures and printing them using an in‐house 3D printing system. The SFY decoction‐based 3D‐printed gels were prepared following a modified version of a previous method. Specifically, 2% and 2.7% high acyl gellan gum (HAG) and gelatin (GL) hydrogels were made by dissolving the powder in a solution at about 50°C under continuous stirring. These hydrocolloids were then mixed with 1%, 2%, 3%, or 4% glycerin to form the final testing gels. An extrusion‐based 3D printer (Luckybot One, Wiiboox Technology Co. Ltd., Nanjing, China) with a syringe and a retractable plunger was operated at around 26°C. A 0.25 mm inner diameter nozzle was used for printing, and images were captured from the top side on a food‐grade glass plate using a mobile phone. Uniform lighting was achieved with a white mini‐studio light box (Rui Teng Digital, Zhejiang, China) containing 144 LED aerial lights above the inks.

### 2.13 Statistical Analysis

All assays were conducted in biological triplicate unless specified otherwise. The means of two independent groups were compared with an unpaired two‐tailed Student's *t*‐test. The normally distributed data among more than two groups were compared via one‐way ANOVA with Tukey's post hoc test. Data were expressed as mean ± standard deviation (SD) and analyzed on GraphPad Prism 9.5.0 (San Diego, CA, USA). The significance levels were *p < 0.05, **p < 0.01, and ***p < 0.001.

## 3 Results and Discussion

### 3.1 Sensory and Fuzzy Comprehensive Evaluation of Optimized Compatibility Components

**FIGURE 1:**
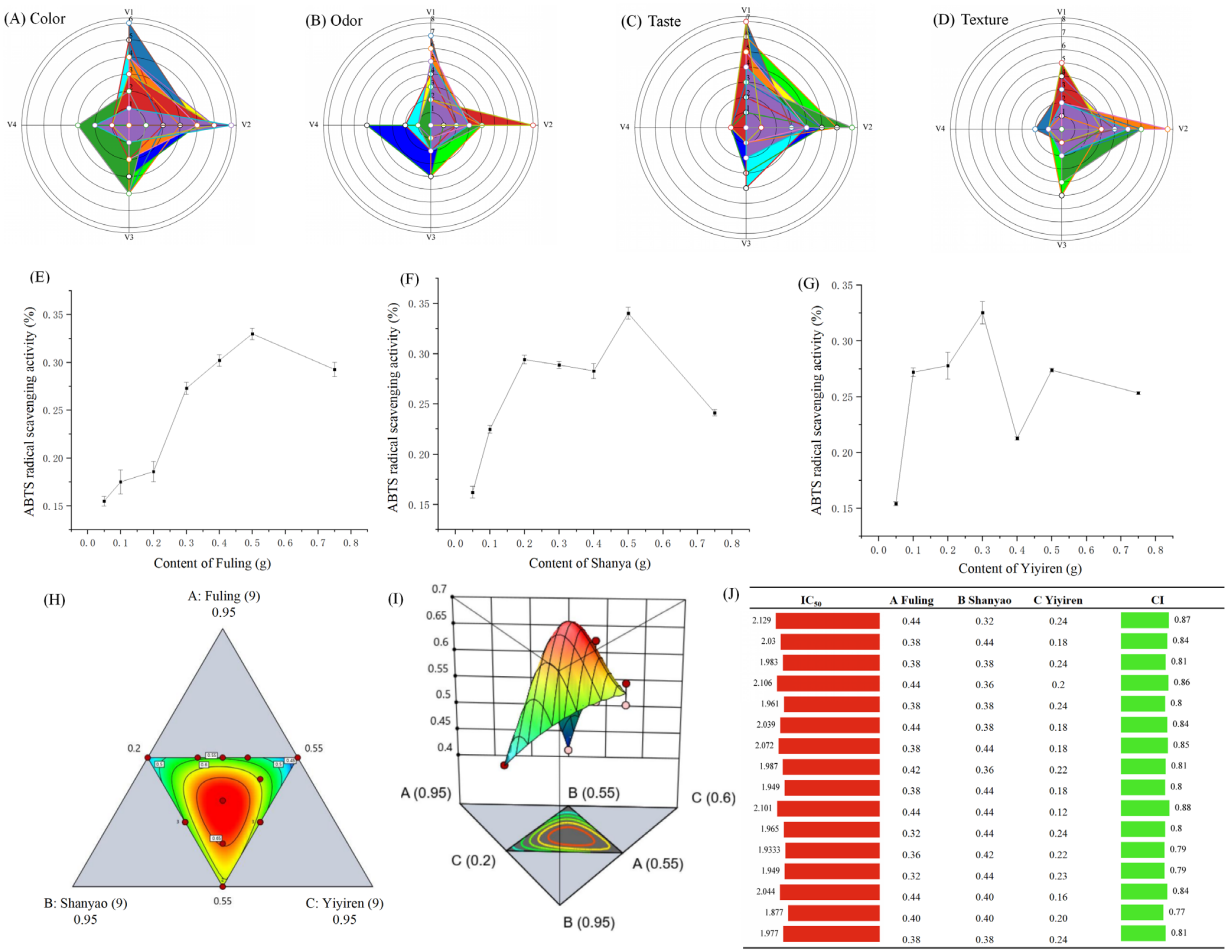
Optimized compound formula compatibility: sensory evaluation (A–D), antioxidant effect (E–G), and component quantification analysis (H–J). Sums of sensory scores for quality attributes of tested samples: *T*_1_ ( *Euryale ferox* , Lotus seed, Shanyao), *T*_2_ ( *Euryale ferox* , Lotus seed, Yiyiren), *T*_3_ ( *Euryale ferox* , Lotus seed, Fuling), *T*_4_ (Lotus seed, Shanyao, Yiyiren), *T*_5_ (Lotus seed, Shanyao, Fuling), *T*_6_ (Shanyao, Yiyiren, Fuling), *T*_7_ ( *Euryale ferox* , Shanyao, Yiyiren), *T*_8_ ( *Euryale ferox* , Shanyao, Fuling), *T*_9_ ( *Euryale ferox* , Yiyiren, Fuling), *T*_10_ (Lotus seed, Yiyiren, Fuling).

To enhance the medicinal value and reduce production costs of Sifang Tang, sensory evaluation and fuzzy mathematical methods were utilized to optimize the active ingredients and compositions. The compatibility of the five components (Qianshi, Shanyao, Fuling, Lianzi, and Yiyiren) in Sifang Tang was analyzed through sensory evaluation (Table 1) and radar plotting (Figure 1A–D). In detail, after the individual sensory attribute for each polyherbal formulation was calculated, the highest color was found on sample 6 (V_1_ = 6), followed by sample 4 (V_1_ = 5), and samples 1, 5, and 7 (V_1_ = 4). As for odor, the highest polyherbal formulation on the response scale was found in sample 6, which linguistically means “Good.” The highest SI on taste, flavor, and mouthfeel was found all on the response scale F4, which linguistically implies “Good.” The SI of color was on the response scale F3, which linguistically refers to “Satisfactory.” For sample 6, the frequency of odor under “Excellent,” “Good,” “Average,” and “Poor” is 7, 2, 1, and 0, and the frequency of odor is 7, 3, 0, and 0 respectively. From the above ranking, in the EFSSE fortified bread sample, sample 6 (Shanyao, Fuling, and Yiyiren) showed better sensory evaluation compatibility.

Traditional sensory evaluation methods are frequently hard to achieve consensus, owing to personal subjective perceptions, environmental variations, and psychological fluctuations. In comparison, membership function theory‐based fuzzy mathematical sensory evaluation can enhance the reliability of sensory evaluations, and thus has been innovatively integrated into food sensory assessment (Pallavi 2025). The sensory evaluation data from the 10 evaluators (Table 1) were aggregated into matrix *R* by dividing the number of votes received for each grade by 10. The weight set *X* was then integrated with matrix *R* to form an evaluation matrix *Y* = *X* × *R*. Then matrix *Y* was processed using a comprehensive scoring matrix *T*. The set of evaluation grades *K* = {90, 70, 50, 30} was utilized, and each grade was multiplied by its corresponding weight and summed to compute the total fuzzy comprehensive evaluation score for each sample. For instance, when the colors of medicinal solutions in the first group were evaluated: four evaluators rated it as excellent, two as good, four4 as average, and none as poor, resulting in *R*_color_ = (0.4, 0.2, 0.4, 0), *R*_odor_ = (0.3, 0.4, 0.2, 0.1), *R*_taste_ = (0.3, 0.5, 0.1, 0.1), and *R*_texture_ = (0.2, 0.6, 0.2, 0). Then we have

$$Y_{1}=R_{1}\times X=\left|\begin{array}{c} \begin{array}{c} 0.4\\ 0.3 \end{array}\begin{array}{c} \;0.2\;\\ \;0.4 \end{array}\;\begin{array}{c} 0.4\\ 0.2 \end{array}\;\begin{array}{c} 0\\ 0.1 \end{array}\\ \begin{array}{c} 0.3\;\\ 0.2 \end{array}\begin{array}{c} 0.5\\ 0.6 \end{array}\begin{array}{c} \;0.1\\ \;0.2 \end{array}\begin{array}{c} \;0.1\\ 0 \end{array} \end{array}\right|\times \left(0.24,0.28,0.33,0.15\right)=\left(0.309,0.415,0.215,0.061\right)$$

Similarly, we have *Y*_2_ = (0.341, 0.33, 0.427, 0.015), *Y*_3_ = (0.108, 0.446, 0.313, 0.133), *Y*_4_ = (0.343, 0.296, 0.266, 0.095), *Y*_5_ = (0.461, 0.429, 0.082, 0.028), *Y*_6_ = (0.616, 0.311, 0.043, 0.03), *Y*_7_ = (0.459, 0.432, 0.109, 0), *Y*_8_ = (0.231, 0.457, 0.212, 0.1), *Y*_9_ = (0.434, 0.422, 0.072, 0.072), and *Y*_10_ = (0.326, 0.435, 0.176, 0.072). Then, we get *T*_1_ = 0.309 × 90 + 0.415 × 70 + 0.215 × 50 + 0.061 × 30 = 69.44, *T*_2_ = 75.59, *T*_3_ = 60.58, *T*_4_ = 65.74, *T*_5_ = 72.41, *T*_6_ = 80.26, *T*_7_ = 77, *T*_8_ = 66.38, *T*_9_ = 74.36, and *T*_10_ = 70.75.

With sensory evaluation of fuzzy mathematical models considered, the polyherbal formulations can be ranked as follows: *T*_6_ > *T*_7_ > *T*_2_ > *T*_9_ > *T*_5_ > *T*_10_ > *T*_1_ > *T*_8_ > *T*_4_ > *T*_3_. After quantitative analysis of sensory evaluation using fuzzy mathematics, the group members/consumers believed sample *T*_6_ (Shanyao, Fuling, and Yiyiren) had higher overall sensory acceptability than the average. Moreover, the overall acceptability parameter was mainly dependent on quantitative analysis of personalized sensory attributes of the samples, including taste, flavor, and texture. These findings indicate the sensory characteristics of the SFY decoction are closely linked to the proportions of Shanyao, Fuling, and Yiyiren, which could be explained by the different molecular profiles of each herb that contribute uniquely to taste and texture. However, the sample scope of the current study was limited to school‐based participants and thus may not fully reflect broader market preferences. Future work will focus on validating the model predictions with large‐scale real‐world consumer datasets to enhance its practical applicability.

### 3.2 Single‐Factor Antioxidant Effect Evaluation

According to Chen et al. (2007), the selected amounts of herbal extracts remarkably impact the performance of antioxidant extracts. Therefore, evaluating the antioxidant capacity of the preliminarily optimized polyherbal formulation is a prerequisite for RSM design. The analysis of ABTS^+^ scavenging activity revealed interesting trends in the antioxidant capacity of the individual components (Figure 1E–G). The antioxidant capacity of Fuling was initially enhanced with the increasing dosage, reaching a peak at 0.5 g. In contrast, the antioxidant capacity of Shan Yao fluctuated, peaking at 0.35 with varying dosages. Yiyiren displayed a peak antioxidant capacity of 0.35 at 0.5 and a low point of 25% at 0.4. These findings allowed determination of the optimal dosage ranges for Shan Yao, Fuling, and Yiyiren, which were utilized as factor level ranges in subsequent RSM design.

### 3.3 Central Composite and Mixture Design Analysis of Ratio and Dosage for Optimizing Compatibility

The design consists of 16 experiments, including replicate experiments to check the consistency of the ABTS scavenging activities. Experimental response data for each experiment and the responses of each run of the testing design were shown in Table 2. The importance of this study and the validity of regression equations were investigated via ANOVA. The special cubic model best interpreted the impact of mixing proportions on ABTS scavenging activity. The quadratic model and the special cubic model were added with backward elimination to remove insignificant terms. The adjusted *R* ^2^ of 0.8558 showed the chosen models were fit. The regression coefficient and ANOVA of the second‐order polynomial models for total antioxidant activity were summarized in Table 3. In the mixture design, the model has a low coefficient of lack‐of‐fit (*p* > 0.05), indicating the model has higher reliability. Simultaneous optimization of herbal mixture proportions showed the optimal formulation was based on the desirability functions of 0.984, 0.879, and 0.878. For validation, these formulations were made and examined using the same assays, and the results were compared with the predictions from the regression model. All the testing data matched well with the results obtained under the optimal extraction conditions, which validates high correlations with the RSM models. This result implies the regression models are significant and predictive. In all, the method can provide consistent results and is suitable for investigating the ABTS scavenging activities of polyherbal formulations.

**TABLE 3:** ANOVA results for the regression model.

Source	Sum of squares	Freedom	Mean square deviation	*F*	*p*	Significance
Model	0.0606	6	0.0101	14.08	0.0004	**
Linear	0.0194	2	0.0097	13.49	0.002	*
AB	0.0154	1	0.0154	21.46	0.0012	*
AC	0.0139	1	0.0139	19.33	0.0017	*
BC	0.0171	1	0.0171	23.89	0.0009	**
ABC	0.0122	1	0.0122	17.05	0.0026	*
Residual	0.0065	9	0.0007			
Lack of fit	0.0024	4	0.0006	0.7269	0.6102	
Pure error	0.0041	5	0.008			
*Y* = −108.15 × A + −111.979 × B + −278.174 × C + 446.071 × AB + 808.139 × AC + 830.832 × BC + −1816.12 × ABC						

Abbreviation: ns, not significant at *p* > 0.05.

* Significant at *p* ≤ 0.05.

** Significant at *p* ≤ 0.01.

### 3.4 Interaction of Polyherbal Formulations on Antioxidant Activities

The effect of herbal mixture on ABTS scavenging activity was demonstrated in a 3D surface plot drawn with the special cubic model. Each point on the plot implies the specific proportion of components in the mixture. Three components (Shanyao, Fuling, and Yiyiren) were included in the 3D surface plot. The ABTS scavenging activity maximized at the area with a ternary mixture of Shanyao, Fuling, and Yiyiren, as reflected on the 3D surface plot, in which the curve moves downward from the vertex to the midpoint. The flat plane on the surface plot is tipped upward to the direction of the intersection point, meaning all herbs contribute to the ABTS scavenging activity. Consistent with the ANOVA, significant interactions (*p* ≤ 0.05) were found between Fuling and Shanyao (AB), Fuling, and Yiyiren (AC), and among Fuling, Yiyiren, and Shanyao (ABC). Shanyao and Yiyiren (BC) were observed as a significant interaction (*p* ≤ 0.01). The elliptical contours in the contour plots indicate that the interaction between two factors is significant. Therefore, the optimal formulation was assessed for ABTS scavenging activity in vitro and finally recommended for industrial applications.

### 3.5 Antioxidant Synergism

To evaluate the interactions in the SFY‐based polyherbal formulations, the overall antioxidant capacities of 16 combinations of extracts and the three constituent herbs (Shanyao, Fuling, and Yiyiren) were assessed. The IC_50_ of Shanyao, Fuling, and Yiyiren was 2.3306, 2.3216, and 2.9349 mg/mL, respectively, and the IC_50_ of each combination was drawn as *X* and *Y* axes. The values above (antagonistic effect) or below (synergistic effect) the line were drawn with both IC_50_ values. The combination of these herbs in the SFY‐formula demonstrated a synergistic effect, resulting in a lower IC_50_ compared to the individual herbs. The CI further validated this synergism, with the CI within 0.77–0.88 (Figure 1J and Table 4). This synergistic effect can be explained by the complementary actions of the bioactive compounds in these herbs. For example, Shanyao contains compounds that enhance the expressions of antioxidant enzymes (Ndouma et al. 2024), whereas Fuling and Yiyiren provide polysaccharides (Qiu et al. 2024) and antioxidant peptides (Igbokwe et al. 2024), respectively, which collectively boost the overall antioxidant capacity. Additionally, the increase in IC_50_ in the combinations led to a larger CI, and a reasonably high correlation between IC_50_ and CI was found (*Y* = 0.4455*x* − 0.0713, *R* ^2^ = 0.9665). To validate this correlation, we tested an independent set of four additional formulations not included in the original design. These formulations were prepared using the same extraction methods but with random variations in herb ratios. The IC_50_ and CI of these new samples were measured, and a similar correlation was observed, confirming the robustness of the relationship. This positive correlation between IC_50_ and CI indicates the compounds work synergistically to enhance free radical scavenging activity, possibly by targeting multiple oxidative stress pathways. The antioxidant capacity of the polyherbal formulation is not merely additive, but instead, the compounds in the mixture interact to produce greater efficacy than would be expected from the individual herbs. The highest radical scavenging activity (67.60% ± 0.60%) was achieved in combination 15, which is also the highest CI of all combinations. The results suggest this particular combination represents the optimal balance of herbs, where the proportions of Shanyao, Fuling, and Yiyiren maximize their combined antioxidant effects. Therefore, the current research on synergistic effects suggests this decoction may be effective.

**TABLE 4:** Synergistic index of SFY‐based polyherbal formulations.

Num.	IC_50_	Proportion of A	Proportion of B	Proportion of C	CI
1	2.129	0.44	0.32	0.24	0.87
2	2.03	0.38	0.44	0.18	0.84
3	1.983	0.38	0.38	0.24	0.81
4	2.106	0.44	0.36	0.2	0.86
5	1.961	0.38	0.38	0.24	0.8
6	2.039	0.44	0.38	0.18	0.84
7	2.072	0.38	0.44	0.18	0.85
8	1.987	0.42	0.36	0.22	0.81
9	1.949	0.38	0.44	0.18	0.8
10	2.101	0.44	0.44	0.12	0.88
11	1.965	0.32	0.44	0.24	0.8
12	1.9333	0.36	0.42	0.22	0.79
13	1.949	0.32	0.44	0.23	0.79
14	2.044	0.44	0.4	0.16	0.84
15	1.877	0.4	0.4	0.2	0.77
16	1.977	0.38	0.38	0.24	0.81

### 3.6 Construction of “Herb‐Compound‐Target” Network

**FIGURE 2:**
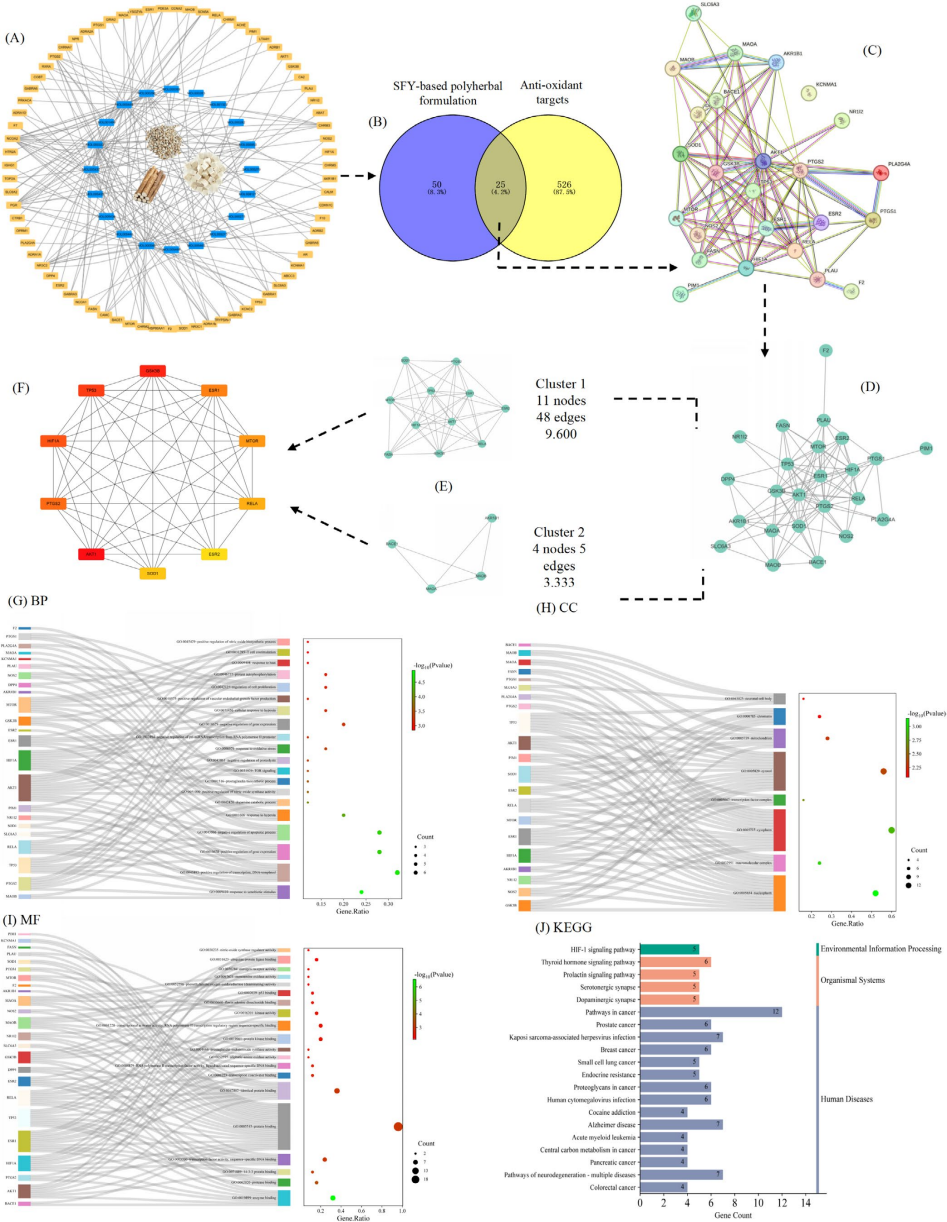
Identification of active compounds (A) and exploration (B–F) and enrichment analysis of key targets in SFY‐formula (G–J). (A) Screening of active compounds and co‐construction of target network for SFY‐formula. (B–F) PPI network clustering analysis of B core targets. (G–I) GO enrichment analysis of biological process (G), cellular component (H), molecular function (I), and KEGG signaling pathway enrichment analysis (J) of potential targets in SFY‐formula.

To further clarify the pharmacological effects of the optimized SFY‐based polyherbal formulation (named SFY‐formula), we investigated the active chemical components and potential targets of SFY‐formula using network pharmacology, and generated a “herb‐compound‐target” network (Figure 2A). The blue and yellow quadrilaterals represent active compounds and potential targets in the network respectively. Based on ADME screening standards (Javed et al. 2024), the associations among 3 herbs, 20 components, and 150 reviewed target genes of SFY‐formula were tested. In detail, there were six species from Shanyao, six species from Fuling, and eight species from Yiyiren. In terms of degree, the top 10 active compounds were stigmasterol (MOL000449), diosgenin (MOL000546), kadsurenone (MOL000322), hederagenin (MOL000296), hancinone C (MOL005430), AIDS180907 (MOL005465), CLR (MOL000953), isofucosterol (MOL005440), sitosterol alpha1 (MOL001323), and sitosterol (MOL000359). These compounds may be critical in defending oxidant damages after the use of SFY‐formula. Additionally, the collected targets after de‐duplication with the highest degrees are NCOA2 (*n* = 17), PDE3A (13), NR3C1 (10), PTGS2 (8), CHRM3 (6), ADRA1B (5), CAMC (5), GABRA1 (5), CHRM2 (4), and NCOA1 (4), which may be the core points in the network and play key roles in defending oxidant damages.

### 3.7 PPI Construction and Subcluster Analysis

Comprehensive data regarding relationships between the overlap targets were merged into the PPI network. Totally 551 reviewed targets were identified from GeneCards, Herb 2.0, and OMIM databases after de‐duplication. Moreover, 75 predicted target genes in the SFY‐based polyherbal formulations were obtained from Section 2.7. Totally 25 genes were identified in a Venn plot as the core targets involved in oxidant resistance, and then inputted into STRING 12.0 to build PPI with a degree cutoff of 2, node score cutoff of 0.2, *K*‐core of 2, and largest depth of 100. The network analysis showed the PPI of intersection consisted of nodes and edges, which represent the targets and interactions of active antioxidant compounds respectively. Statistically, the PPI network contains totally 25 nodes and 99 interactive associations with a mean local clustering coefficient of 0.74 and a mean node degree of 7.92 with the PPI enrichment *p* < 4.5e‐14. As reported, nodes with plentiful edges may play a more significant role in the network and those on which to focus. As one of the core targets (Figure 2C), AKT1 is the most relevant target ranked by the degree, followed by GSK3B, TP53, HIF1A, PTGS2, ESR1, MTOR, RELA, SOD1, and ESR2, which may be critical in avoiding oxidant damages after the use of SFY‐formula.

To further investigate the target interaction of PPI, the clusters for the core targets were constructed using the plugin MCODE. Two clusters within the PPI network were identified (Figure 2D–F). Cluster 1 with the highest score has 11 nodes and 48 edges, including SOD1, PTGS2, TP53, MTOR, ESR1, HIF1A, AKT1, ESR2, FASN, GSK3B, and RELA. A strong relationship between HIF1A or GSK3B and cervical cancer was reported before. Ren et al. (2013) found that cancer‐associated fibroblasts secreted high‐level IL‐6 with activated STAT3, and were senescent at early passages in culture or in cervical cancer tissues infected with high‐risk human papilloma virus. Cluster 2 with the second highest score consists of four nodes and five edges, including BACE1, MAOA, MAOB, and AKR1B1.

### 3.8 GO/KEGG Enrichment Analyses to Understand Antioxidant Mechanism of Compounds

The oxidant damage‐related targets were characterized through GO enrichment analysis to completely clarify the potential antioxidant mechanism of SFY‐formula. The top remarkably enriched items of BP, CC, and MF were selected based on *p* values (Figure 2G–I), which represent the degree of enrichment. A redder dot indicates a lower *p* value and greater enrichment. Pathway enrichment analysis of targets showed the targets were major components of the following BP‐associated pathways: positive modulation of NO biosynthesis (GO:0045429), T cell costimulation (GO:0031295), response to heat (GO:0009408), protein autophosphorylation (GO:0046777), control of cell proliferation (GO:0042127), positive modulation of VEGF generation (GO:0010575), cell response to hypoxia (GO:0071456), negative control of gene expression (GO:0010629), negative control of pri‐miRNA transcription from RNA polymerase II promoter (GO:1902894), and reaction to oxidative stress (GO:0006979). In CC enrichment processes, the principal pathways were related to neuronal cell body (GO:0043025), chromatin (GO:0000785), mitochondrion (GO:0005739), cytosol (GO:0005829), transcription factor complex (GO:0005667), cytoplasm (GO:0005737), macromolecular complex (GO:0032991), and nucleoplasm (GO:0005654). GO analysis revealed the MFs related to protein kinase binding (GO:0019901), transcriptional activator activity, RNA polymerase II transcription regulatory zone sequence‐specific binding (GO:0001228), kinase activity (GO:0016301), flavin adenine dinucleotide binding (GO:0050660), p53 binding (GO:0002039), phenethylamine:oxygen oxidoreductase (deaminating) activity (GO:0052596), monoamine oxidase activity (GO:0097621), (GO:0030284), estrogen receptor activity ubiquitin protein ligase binding (GO:0031625), and NO synthase regulator activity (GO:0030235).

The KEGG pathways of SFY‐formula were investigated to further understand the antioxidant damage mechanism of the compounds. There were 172 significantly related pathways, and the top 20 KEGG pathways retrieved based on *p* values were displayed in Figure 2J. The KEGG analysis illustrates the main pathways can be roughly divided into environmental information processing, organismal systems, and human diseases. Notably, the HIF‐1 signaling pathway is the most unregulated pathway in environmental information processing and is closely related to antioxidant mechanisms. Additionally, the thyroid hormone signaling pathway, prolactin signaling pathway, serotonergic synapse, and dopaminergic synapse from organismal systems may be the key antioxidant mechanisms. In total, SFY‐formula may resist oxidant damages by influencing the above pathways.

### 3.9 Analysis of Affinity Docking Between Active Ingredients and Potential Targets

**FIGURE 3:**
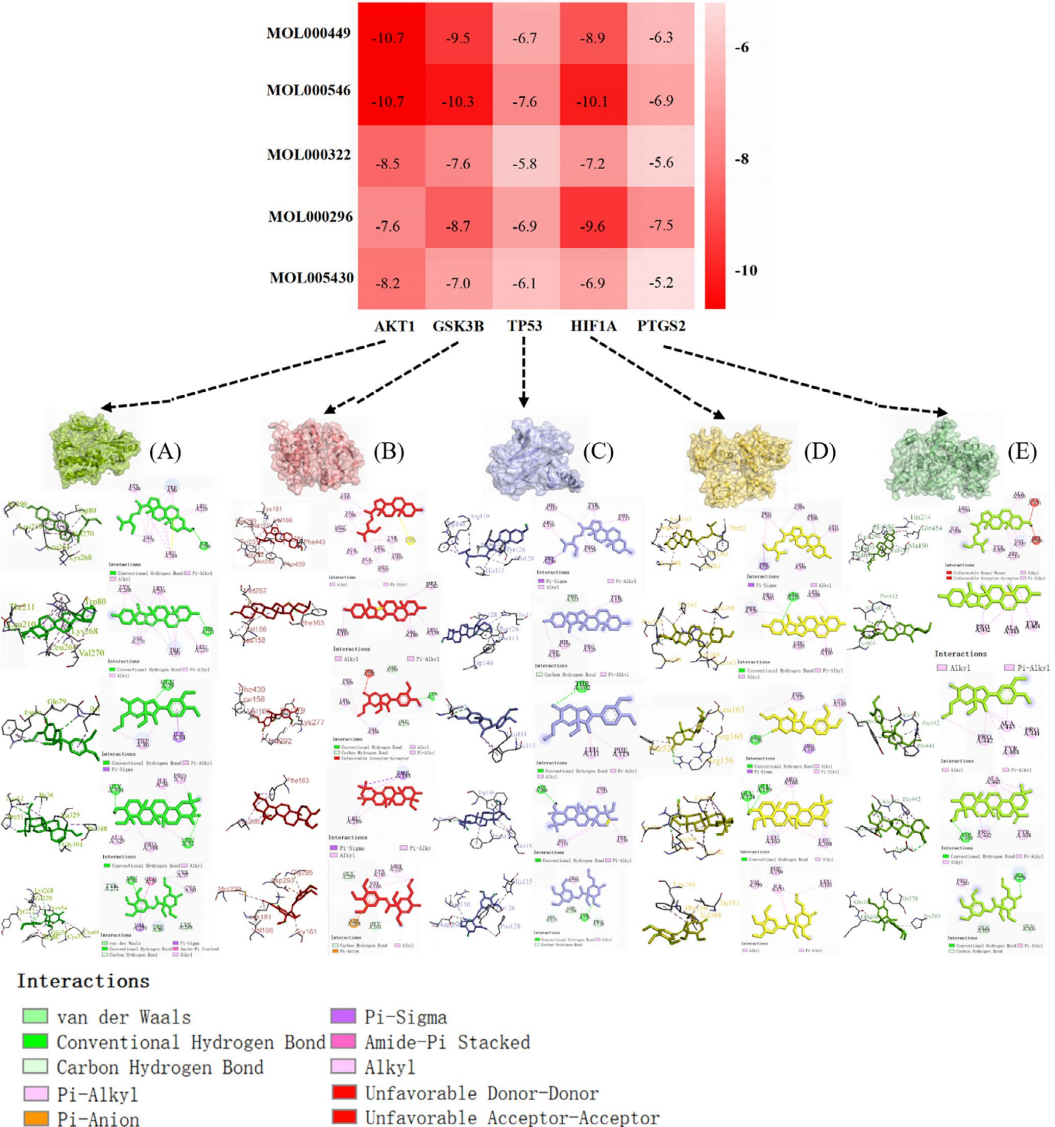
Molecular docking and binding affinities of top targets and core ingredients with AKT1 (A), GSK3B (B), TP53 (C), HIF1A (D), and PTGS2 (E).

In silico molecular docking was performed to profoundly and systematically understand the interaction mechanism between core targets and ingredients, which accord with the degrees in the network analysis (Hou et al. 2024). Based on the core subnetwork, the top five targets (AKT1, GSK3B, TP53, HIF1A, and PTGS2) were selected as the receptors for docking with the core ingredients (a, b, and c). As reported, a more negative binding energy reflects that the chemical constituents and the targets bind more spontaneously without consuming energy. Therefore, the constituents with a binding energy of −6.0 kcal/mol or less were chosen as the basis to screen potential targets. Most of the binding capacities between core ingredients and targets were less than −6.0 kcal/mol (Figure 3), indicating that chemical constituents and targets can spontaneously bind and further confirming the results of the network pharmacological analysis (Zhao et al. 2024). The 3D docking conformations and 2D diagrams in Figure 3 display the optimal results of energy interaction between active compounds and core targets. In general, various interaction forces exist between active compounds and core targets, primarily including hydrogen bonding, pi‐pi stacking, pi‐alkyl, pi‐donor hydrogen bond, pi anion, pi‐sigma, and van der Waals forces. Further analysis of the 2D binding patterns revealed that the binding forces of pi‐pi stacking, pi‐alkyl, pi anion, and pi‐sigma mainly occurred in the phenyl rings of the compounds. The involved amino acids in AKT1 were mainly VAL A270, LYS A268, LEU A264, LEU A260, and TRP A80.

### 3.10 SFY‐Formula Enhances Peroxidase Activity and Resists Oxidants by Regulating AKT1/GSK3β/HIF1α Pathway

**FIGURE 4:**
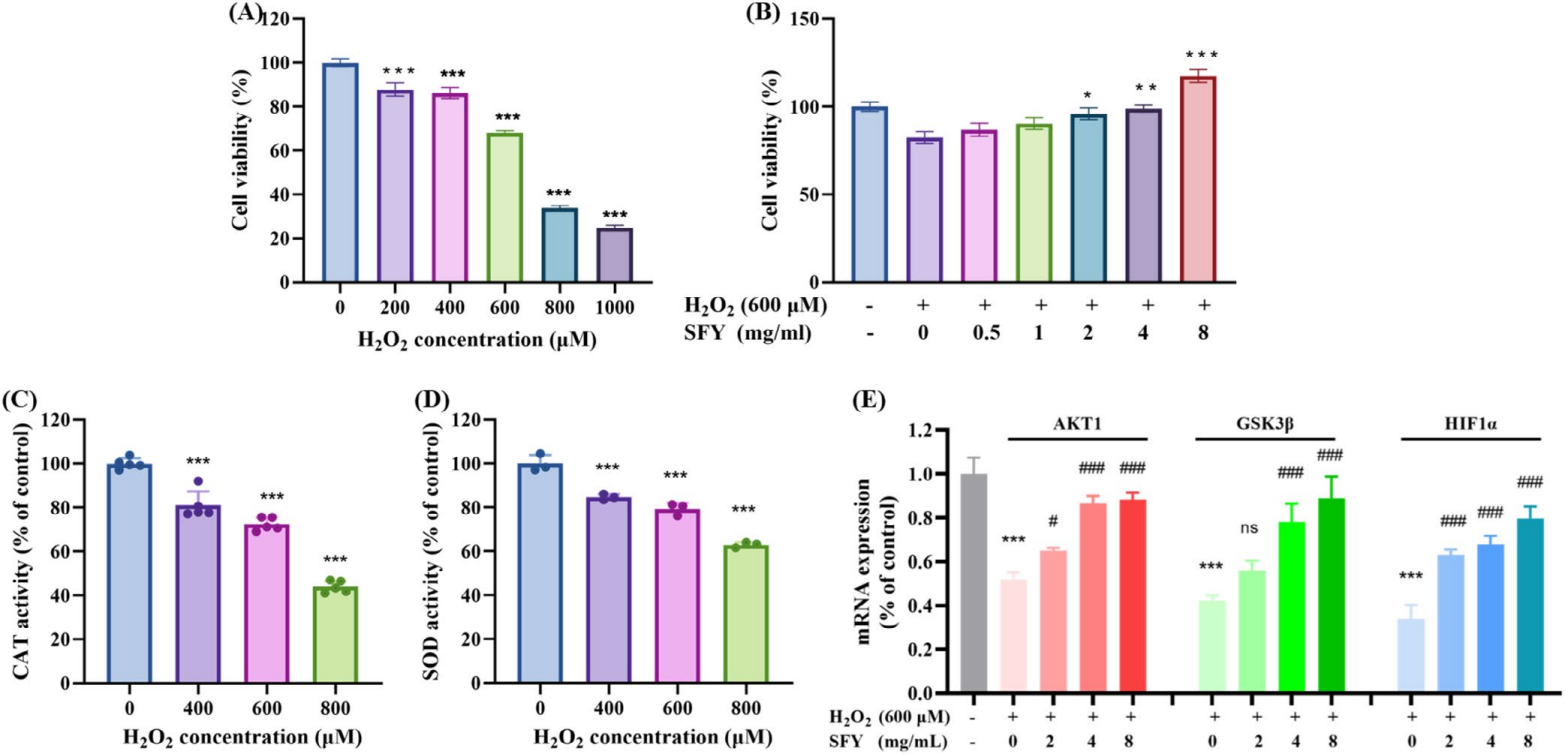
SFY‐formula enhances peroxidase activity and exerts antioxidant effects by regulating the AKT1/GSK3β/HIF1α pathway. (A) The viability of BNLCL.2 cells was assessed using the MTT assay after exposure to varying concentrations of H_2_O_2_ (0–1000 μmol/L). (B) The protective effects of SFY‐formula (0–8 mg/mL) on BNLCL.2 cells pretreated with 600 μM H_2_O_2_. (C) CAT and (D) activities were assessed in BNLCL.2 cells pretreated with 600 μM H_2_O_2_ for 4 h and then co‐cultured with various concentrations of SFY‐formula (0, 2, 4, and 8 mg/mL) for 24 h. (E) SFY‐formula regulates the expression of AKT1, GSK3β, and HIF‐1‐α in BNLCL.2 cells. Effect of co‐incubation of BNLCL cells with different concentrations of SFY‐formula (0, 2, 4, and 8 mg/mL) on the relative expression of AKT1, GSK3β, and HIF‐1‐α after advance pretreatment with 600 μM H_2_O_2_. Statistical significance is denoted as *p < 0.05, **p < 0.01, and ***p < 0.001.

To test the protective effects of SFY‐formula on cell viability in the presence of H_2_O_2_, BNLCL.2 cells were treated with 0, 200, 400, 600, 800, or 1000 μmol/L H_2_O_2_ for 24 h (Nogueira‐Pedro et al. 2013). H_2_O_2_ caused dose‐dependent cytotoxicity and cell death, and 600 and 800 μmol/L H_2_O_2_ induced oxidative damage in 31.71% and 66.23% of the cells, respectively (Figure 4A). Then, 600 μmol/L was chosen as the optimal culture concentration in further experiments. BNLCL.2 cells were pretreated with 600 μmol/L H_2_O_2_ and then co‐cultured with 0–8 mg/mL SFY‐formula. Compared to the control group, cell viability significantly increased after SFY‐formula treatment. SFY‐formula at 2–8 mg/mL effectively reduced H_2_O_2_‐induced cytotoxicity and promoted cell proliferation (Figure 4B). As a result, SFY‐formula reversed the potent cytotoxic effects of H_2_O_2_ on BNLCL.2 cells.

The oxidizing effect of H_2_O_2_ and the antioxidant effect of SFY‐formula were investigated by assessing SOD and CAT activities in BNLCL.2 cells (Figure 4C,D). The CAT and SOD activities in BNLCL.2 cells exposed to 400, 600, and 800 μM H_2_O_2_ were significantly and dose‐dependently reduced compared to the control. In further experiments, 600 μM was selected as the optimal culture condition and used in co‐culture with SFY‐formula (0, 2, 4, and 8 mg/mL). CAT levels in the BNLCL.2 cells exposed to 600 μM H_2_O_2_ were inhibited by 52.88% compared to the control, which was significantly increased by 66.72%, 99.79%, and 114.07% by SFY‐formula, respectively (Figure 4C). These results indicate the antioxidant ability of SFY‐formula can reverse the oxidative damages caused by H_2_O_2_ exposure. We also evaluated the SOD activity in vitro after the administration of SFY‐formula (Figure 4D). The similar results further reveal that SFY‐formula can be an antioxidant drug against H_2_O_2_‐induced oxidative damages.

Furthermore, the potential targets of AKT1, GSK3β, and HIF‐1‐α in the SFY‐formula were preliminarily identified through network pharmacological analysis and molecular docking. To further confirm their regulatory roles, we examined the impacts of H_2_O_2_ and SFY‐formula on the expressions of relevant genes using qRT‐PCR. H_2_O_2_ induced BNLCL.2 cells to significantly downregulate the expressions of oxidative stress‐related genes (AKT1, GSK3β, and HIF‐1‐α), whereas different concentrations of SFY‐formula dose‐dependently reversed gene expressions, which are consistent with the network screening results (Skuli et al. 2006; Figure 4E). qRT‐PCR results further verify that SFY‐formula can regulate the AKT1/GSK3β/HIF‐1‐α signaling axis to exert antioxidant protection. As reported by Qi et al. (2023), activated AKT/GSK3β signaling maintains elevated HIF1α levels, exemplifying the intrinsic cross‐talk among these pathways that orchestrate cellular adaptive responses. However, there is no direct mechanistic evidence, since no Western blot validation was done for the AKT1/GSK3β/HIF1α signaling axis. Future research will focus on filling this gap.

### 3.11 Application and Evaluation of 3D Printing in SFY‐Formula

**FIGURE 5:**
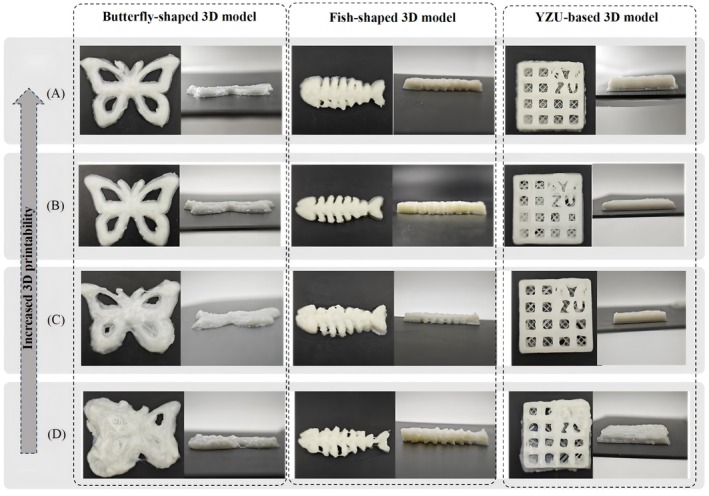
Representative elevation view of 3D‐printed geometries made with different formations.

Shape fidelity, one effective method to measure the quality of a printed structure, implies the printability of a hydrogel (Guan, Ren, et al. 2024). For one‐layered grids, a printable hydrogel shall constitute a structure with a high shape fidelity, where the grids and square holes must accord with the predesigned pattern. In contrast, a hydrogel with low printability will form an abnormal shape, where circular‐like holes and irregular grids exist in a printed grid (Ahmadzadeh et al. 2023). Thus, the best concentration of each hydrogel for printing can be clarified by examining the shape fidelity of a printed structure (Nguyen et al. 2025). A 3 × 3 grid of 12‐mm squares was laid over the photographs to demonstrate the intended dimensions of the triple‐steps, and thus helped qualify any deviation in the dimensions of the prints away from them. The front‐elevation views of the optimal printing for extruding hydrogels with various GL concentrations were recorded in Figure 5. For the printed inks with high‐GL concentration (Figure 5C,D), the printed filaments were irregular and fractured with a rough surface, and excessive spreading was observed. The irregularity observed in high‐GL gels can be attributed to their higher viscosity and slower flow, which make the extrusion more challenging, leading to inconsistency in filament deposition and surface texture. Differently, for the printed inks with low GL concentration (Figure 5A), the printed filaments were irregular with the highest printability. This lower concentration likely allowed for smoother extrusion but may have resulted in less structural integrity, as the filaments lacked the strength to hold shape after extrusion, thus compromising the print fidelity. The top view of the dual cross‐point cubical 3D printed structures of the C50G50 gels showed the stable printed layers in the initial design with high resolution from 2, 4 to 6 layers with defined pores in the constructs (Figure 5B). Therefore, the best content for printing gelatin hydrogels was 8 wt%. Given that the SFY formulation contains bioactive components capable of modulating oxidative stress pathways that are closely associated with HCC, this ink holds great potential for further development into a diagnostic platform. Certainly, further research is needed to fully validate such a possibility.

## 4 Conclusions

The classic formulation of Ming Fang Si Wu Tang was optimized using sensory indexes and fuzzy mathematical models to enhance its antioxidant efficacy and align it with consumer preferences, aiming to reduce production costs. The combination of Shanyao, Fuling, and Yiyiren was identified as the optimal formulation (SFY‐formula), which demonstrated the highest antioxidant activity and a CI of 0.79 at the Shanyao:Fuling:Yiyiren ratio of 2:2:1. Network pharmacology and molecular docking analyses revealed that the active ingredients in the SFY‐formula interact with key targets (AKT1, GSK3B, TP53, HIF1A, and PTGS2). Experimental validation using H_2_O_2_‐induced BNLCL.2 hepatocyte cells confirms that the SFY‐formula enhances peroxidase activity by modulating the AKT1/GSK3β/HIF1α signaling pathway, thereby exerting protective antioxidant effects. Notably, molecular docking and cell‐based assays jointly confirm the crucial roles of AKT1, GSK3β, and HIF1α, underscoring their significance in preventing oxidative damages. GL‐based 3D printing inks were successfully developed and utilized to create customized, soft, multinutrient, and multicomplex antioxidant synergistic functional foods. This approach exemplifies the potential of integrating 3D printing technology with traditional formulations to produce functional foods tailored to individual nutritional needs. Collectively, these findings offer valuable insights into the antioxidant mechanisms of the SFY‐formula and highlight the feasibility of employing 3D printing technology in developing personalized nutrition solutions. The research paves the way for future studies to explore the clinical applications of the SFY‐formula and the broader potential of 3D‐printed functional foods in promoting health and well‐being.

## Author Contributions

**Haoran Fan:** conceptualization, methodology, software, validation, formal analysis, investigation, resources, data curation, writing – original draft, visualization. **Cai You:** methodology, investigation. **Chenxi Ren:** methodology, investigation. **Xiaoxiao Liu:** conceptualization, writing – review and editing, project administration. **Yining Feng:** conceptualization, writing – review and editing, project administration. **Ganghui Chu:** writing – review and editing, supervision, project administration. **Abdulla Yusuf:** writing – review and editing, supervision, project administration. **Chunbo Liu:** resources. **Liemin Ruan:** resources. **Jia Xu:** supervision, project administration. **Tianzhu Guan:** investigation, writing – review and editing, supervision, project administration.

## Conflicts of Interest

The authors declare no conflicts of interest.

## Supporting information


Tables S1–S3

## Data Availability

All data supporting the findings of this study are available within the paper and within its Supporting Information published online.
