# Supplementary material for: Exploring Antioxidant Synergism Mechanism of 3D Printing Based Shanyao–Fuling–Yiyiren Decoction via Fuzzy Mathematical Method, Network Pharmacology, and In Vitro Experimental Validation
Source: Food Sci Nutr. 2025 May 30;13(6):e70349. doi: 10.1002/fsn3.70349 (PMC12124989; doi:10.1002/fsn3.70349)
Supplement: Supplementary file 1 — Tables S1–S3 [file FSN3-13-e70349-s001.docx]

| **SUPPLEMENTARY TABLE 1 Standard of sensory evaluation.** | | |  |  |
| --- | --- | --- | --- | --- |
| **Sensory quality attributes** | **Sensory scale factors** | | | |
|  | **V1** | **V2** | **V3** | **V4** |
|  | **Excellent (80~100)** | **Good (60~79)** | **Average （40~59）** | **Poor （0~39）** |
| **Color** | Brownish-yellow with a glossy surface | Brownish-yellow with a relatively glossy surface | Uneven color, too dark or too light | Dull in color, blackened or very pale |
| **Odor** | Distinct, rich, and well-balanced herbal aroma | Moderately distinct herbal aroma | Faint but acceptable herbal aroma | Lacks the characteristic herbal aroma |
| **Taste** | Pleasant taste, harmonious texture, good palatability | Mild taste, fairly harmonious texture, fairly good palatability | Ordinary taste, unharmonious texture, moderate palatability | Bitter taste with off-flavors, unharmonious texture, poor palatability |
| **Texture** | Clear and transparent, no solid particles or impurities | Clear but opaque, no solid particles or impurities | Slightly cloudy, few solid particles, no impurities | Cloudy and opaque, many solid particles and some impurities |

| **SUPPLEMENTARY TABLE 2 The weight distribution of each factors** | | | | | |
| --- | --- | --- | --- | --- | --- |
| **Evaluation index** | **Score** | | | | |
|  | **Color** | **Odor** | **Taste** | **Texture** | **Total** |
| **Color** | 10 | 4 | 3 | 7 | 24 |
| **Odor** | 6 | 10 | 4 | 8 | 28 |
| **Taste** | 7 | 6 | 10 | 10 | 33 |
| **Texture** | 3 | 2 | 0 | 10 | 15 |

| **SUPPLEMENTARY TABLE 3 Primer sequences** | | | |  |
| --- | --- | --- | --- | --- |
| **Gene** | **Species** | **Forward primer (5'→3')** | **Reverse primer (5'→3')** |  |
| **Akt1** | Mus | TGTTTCTACTGTGGGCAGCA | TGGTCGCGTCAGTCCTTAAT |  |
| \| **GSK3** \| \| --- \| \| | Mus | GCTGGAGTACACACCTACCG | GGAGGGATAAGGATGGTGGC |  |
|  |  |  |  |  |
| **HIF1α** | Mus | GGTTCCAGCAGACCCAGTTA | ATGCCTTAGCAGTGGTCGTT |  |
| **GAPDH** | Mus | CGATGCCCCCATGTTTGTGA | GAGCCCTTCCACAATGCCAA |  |
